# Evaluating the Managerial Feasibility of an AI-Based Tooth-Percussion Signal Screening Concept for Dental Caries: An In Silico Study

**DOI:** 10.3390/diagnostics16040638

**Published:** 2026-02-22

**Authors:** Stefan Lucian Burlea, Călin Gheorghe Buzea, Irina Nica, Florin Nedeff, Diana Mirila, Valentin Nedeff, Lacramioara Ochiuz, Lucian Dobreci, Maricel Agop, Ioana Rudnic

**Affiliations:** 1 Dentoalveolar Surgery, Faculty of Medicine, University of Medicine and Pharmacy “Grigore T. Popa” Iași, 700115 Iasi, Romania; lucianburlea@yahoo.com; 2 National Institute of Research and Development for Technical Physics, IFT Iași, 700050 Iasi, Romania; calinb2003@yahoo.com; 3 “Prof. Dr. Nicolae Oblu” Clinical Emergency Hospital Iași, 700309 Iasi, Romania; 4 Department of Odontology-Periodontology, Fixed Prosthesis, “Grigore T. Popa” University of Medicine and Pharmacy, 700115 Iasi, Romania; irina.nica@umfiasi.ro; 5 Department of Environmental Engineering, Mechanical Engineering and Agritourism, Faculty of Engineering, “Vasile Alecsandri” University of Bacau, 600115 Bacau, Romaniavnedeff@ub.ro (V.N.); m.agop@yahoo.com (M.A.); 6 Department of Odontology-Periodontology, Grigore T. Popa University of Medicine and Pharmacy Iasi, 700115 Iasi, Romania; lacramioara.ochiuz@umfiasi.ro; 7 Department of Physical and Occupational Therapy, “VasileAlecsandri” University of Bacau, 600115 Bacau, Romania; lucian.dobreci@ub.ro; 8 Academy of Romanian Scientists, 3 Ilfov, 050044 Bucharest, Romania; 9 Department of Odontology-Periodontology, Faculty of Medicine, University of Medicine and Pharmacy “Grigore T. Popa” Iași, 700115 Iasi, Romania; ioana.rudnic@umfiasi.ro

**Keywords:** dental caries, mass screening, decision support systems, clinical, artificial intelligence, signal processing, computer-assisted, computer simulation, preventive dentistry, health services administration

## Abstract

**Background:** Early detection of dental caries is essential for effective oral health management. Current diagnostic workflows rely heavily on radiographic imaging, which involves infrastructure requirements, workflow coordination, and resource considerations that may limit frequent use in high-throughput or resource-constrained settings. These contextual factors motivate exploration of adjunct screening concepts that could support front-end triage decisions within existing care pathways. This study evaluates, in simulation, whether modeled tooth-percussion response signals contain sufficient discriminative information to justify further translational and managerial investigation. Implementation costs, workflow optimization, and economic outcomes are not evaluated directly; rather, the objective is to assess whether the technical preconditions for a potentially scalable screening concept are satisfied under controlled in silico conditions. **Methods:** An in silico model of tooth percussion was developed in which enamel, dentin, and pulp/root structures were represented as a simplified layered mechanical system. Impulse responses generated from simulated tapping were used to compute the modeled surface-vibration response (enamel-layer displacement), which served as a proxy for a measurable percussion-related signal (e.g., contact vibration), rather than a recorded acoustic waveform. Carious conditions were simulated through depth-dependent reductions in stiffness and effective mass and increases in damping to represent enamel and dentin demineralization. A synthetic dataset of labeled simulated signals was generated under varying structural parameters and measurement-noise assumptions. Machine-learning models using Mel-frequency cepstral coefficient (MFCC) features were trained to classify healthy teeth, enamel caries, and dentin caries at a screening (triage) level. **Results:** Under baseline simulation conditions, the classifier achieved an overall accuracy of 0.97 with balanced macro-averaged F1-score (0.97). Misclassifications occurred primarily between healthy and enamel-caries categories, whereas dentin-caries cases were most consistently identified. When measurement noise and structural variability were increased, performance declined gradually, reaching approximately 0.90 accuracy under the most challenging simulated scenario. These results indicate that discriminative information is present within the modeled signals at a screening (triage) level, meaning that higher-risk categories can be distinguished probabilistically rather than with definitive diagnostic certainty. Sensitivity and specificity trade-offs were not optimized in this study, as the objective was to assess separability rather than to define clinical decision thresholds. **Conclusions:** Within the constraints of the in silico model, simulated tooth-percussion response signals demonstrated discriminative patterns between healthy, enamel caries, and dentin caries categories at a screening (triage) level. These findings establish technical plausibility under controlled simulation conditions and support further investigation of percussion-based screening as a potential adjunct to clinical assessment. From a healthcare management perspective, the present results address a prerequisite question—whether such signals contain sufficient information to justify translational research, rather than demonstrating workflow optimization, cost reduction, or system-level impact. Clinical validation, threshold optimization, and implementation studies are required before managerial or operational benefits can be evaluated.

## 1. Introduction

Dental caries is among the most prevalent chronic conditions globally and continues to generate a substantial burden for healthcare systems [1]. From a management perspective, its impact extends beyond clinical outcomes to include significant costs associated with diagnosis, treatment, and long-term disease management. Early identification of carious lesions is therefore a key operational objective, as delayed detection often leads to more complex interventions, higher treatment costs, and increased strain on healthcare resources [2].

Current diagnostic workflows in dentistry rely primarily on clinical examination supported by radiographic imaging. While effective, radiographic methods introduce economic, operational, and organizational constraints. These include capital investment in imaging equipment, maintenance and regulatory compliance costs, reliance on trained personnel, and workflow bottlenecks in high-volume clinical environments [3,4,5]. In addition, concerns regarding cumulative radiation exposure have encouraged more conservative use of imaging, particularly in screening and preventive contexts, further complicating decision-making at the organizational level [6].

These challenges are especially pronounced in settings characterized by limited resources or high service demand, such as public dental programs, rural clinics, mobile care units, and community-based screening initiatives [7,8]. In such environments, access to radiographic infrastructure may be restricted, and the need for scalable, low-cost screening mechanisms becomes a critical management concern. Consequently, there is increasing interest in alternative approaches that can support early risk stratification and referral decisions without imposing substantial additional costs or infrastructure requirements.

Within healthcare management research, decision-support technologies have emerged as a means of improving efficiency, standardizing assessment, and optimizing resource allocation across care pathways [9,10,11]. Importantly, such tools are not intended to replace clinical diagnosis but to assist in triage, prioritization, and workflow coordination, particularly at the front end of care delivery. Low-cost, non-invasive screening tools are therefore of particular relevance, as they offer the potential to expand access to preventive services while reducing dependency on resource-intensive diagnostics [12].

Tooth percussion is a routine component of dental examination and is widely used in a qualitative manner to assess tooth condition. Despite its ubiquity, percussion remains a subjective practice and has not been systematically developed into an objective decision-support mechanism. Advances in data-driven health technologies raise the possibility that information embedded in routine clinical interactions could be leveraged to support managerial objectives such as early screening, task shifting, and workflow efficiency [13,14]. Before such approaches can be considered for organizational adoption, however, their feasibility must be evaluated in a controlled and cost-effective manner.

From a biomechanical perspective, tooth percussion induces a transient vibrational response governed by the combined stiffness, mass distribution, and damping characteristics of enamel, dentin, and supporting structures. Demineralization associated with caries reduces mineral content and alters microstructural integrity, which in turn affects effective stiffness and energy dissipation properties of the involved layer. In mechanical systems, such parameter changes can modify natural frequencies, decay rates, and spectral energy distribution of impulse responses. Although the magnitude of these effects in vivo is uncertain, this physical relationship provides a plausible basis for investigating whether depth-dependent structural degradation could produce distinguishable patterns in percussion-derived signals under controlled conditions.

In silico modeling provides a practical means of conducting early-stage feasibility assessments for healthcare technologies without the ethical, logistical, and financial constraints associated with clinical data collection [15,16]. From a management standpoint, in silico studies allow decision-makers to assess whether a proposed approach warrants further investment, pilot testing, or clinical validation. They are particularly well suited for evaluating robustness under variable conditions and for exploring scalability prior to deployment.

While the concept is framed within a healthcare management perspective, the present study does not evaluate clinical decision thresholds, workflow modeling, or cost-effectiveness. Instead, it addresses a prerequisite managerial question: whether tooth-percussion response signals, under controlled simulation conditions, contain sufficient discriminative information to justify further translational and operational investigation.

A critical question is whether caries-related structural changes would meaningfully influence the percussion response in the presence of other clinical confounders. Tooth vibration characteristics in vivo are affected not only by enamel and dentin properties, but also by restorations, cracks, endodontic treatment status, crown materials, occlusal contacts, periodontal ligament compliance, alveolar bone support, and tooth type. These factors may introduce variability that could obscure subtle demineralization-related changes. The present study does not assume that caries would dominate all such influences in clinical recordings. Instead, it isolates the mechanical contribution of depth-dependent structural degradation within a simplified model to determine whether discriminative signal patterns exist under controlled conditions. Establishing this technical separability is a prerequisite step before evaluating how such signals interact with broader clinical variability in real-world settings.

From a healthcare management research perspective, technology assessment typically proceeds in stages: (1) technical feasibility under controlled conditions, (2) empirical validation in real-world environments, (3) operational modeling of workflow integration and resource utilization, and (4) economic evaluation such as cost-effectiveness or budget-impact analysis. The present study addresses only the first stage of this progression. No workflow simulation, triage-utility modeling, resource-allocation analysis, or economic evaluation is performed. Instead, the objective is to determine whether sufficient discriminative signal structure exists to justify subsequent operational and economic investigation.

Accordingly, the objective of this study is to evaluate, within a controlled in silico framework, whether tooth-percussion response signals exhibit discriminative patterns consistent with depth-dependent structural degradation associated with caries. Rather than addressing diagnostic replacement, this work focuses on whether such an approach could complement existing dental workflows by supporting early screening, prioritization, and efficient resource utilization. The findings are intended to inform future clinical validation efforts and to contribute to broader discussions on scalable and accessible technologies for oral health management [17].

In this context, the contribution of the present work is not primarily clinical accuracy, but the evaluation of a technical precondition relevant to healthcare management. Specifically, the study assesses whether simulated tooth-percussion response signals exhibit sufficient discriminative structure to justify subsequent operational modeling, workflow analysis, and economic evaluation. The proposed screening concept is therefore positioned as a potential future component of managerial decision-support frameworks, rather than as a validated operational system.

The remainder of this paper is organized as follows. Section 2 describes the in silico study design, including the mechanical modeling of tooth percussion, the simulation of carious conditions, the generation of synthetic acoustic data, and the machine-learning framework used for screening-level classification. Section 3 presents the results of the in silico experiments, focusing on the characteristics of the simulated signals, classification performance under baseline conditions, and robustness to noise and anatomical variability. Section 4 discusses the findings from a healthcare management perspective, interpreting the results in terms of decision support, workflow integration, resource optimization, scalability, and adoption considerations, and situating the proposed approach within the context of existing screening and diagnostic methods. Finally, Section 5 summarizes the main conclusions and outlines directions for future clinical validation and implementation-oriented research.

## 2. Materials and Methods

### 2.1. Study Design and Scope

This study was designed as an in silico proof-of-concept investigation to evaluate whether tooth percussion acoustics contain sufficient information to support screening-level decision support for dental caries. The methodological goal was not to replicate patient-specific anatomy or to propose a clinical diagnostic replacement, but to assess feasibility, robustness, and scalability under controlled and reproducible conditions.

An in silico approach was selected to:

1. Enable systematic variation in structural and noise parameters,
2. Generate large labeled datasets without ethical or logistical constraints,
3. Evaluate robustness prior to any ex vivo or clinical validation.

The null hypothesis of this study was that simulated tooth percussion acoustic signals do not contain sufficient discriminative information to differentiate between healthy teeth, enamel caries, and dentin caries at a screening level within the defined in silico framework.

The methodological pipeline consisted of:

1. Physics-based simulation of tooth percussion,
2. Synthetic dataset generation with controlled variability,
3. Acoustic feature extraction,
4. Machine-learning-based screening classification,
5. Robustness analysis under increasing uncertainty.

For clarity, all classification outputs produced in this study are interpreted strictly at the screening and triage level. The model is not intended to provide patient-specific diagnoses or to replace established diagnostic procedures, but rather to support early decision-making regarding referral, prioritization, and the selective use of confirmatory imaging.

In this manuscript, the term “signal” refers to the simulated tooth response produced by the mechanical model. No real audio or in vivo recordings were collected. Accordingly, the present study should be interpreted as a simulation-based feasibility analysis of signal separability and robustness, not as an evaluation of a deployed screening device.

No human participants, extracted teeth, or clinical recordings were involved in this study; all data were synthetically generated within the simulation framework.

### 2.2. Mechanical Modeling of the Tooth

#### 2.2.1. Layered Lumped-Parameter Representation

The tooth was modeled as a one-dimensional layered mechanical system, representing enamel, dentin, and pulp/root structures. Each layer was represented by a lumped mass–spring–damper element, capturing inertial, elastic, and dissipative properties.

This abstraction reflects the clinical reality that:

- Enamel is stiff and weakly damped,
- Dentin exhibits lower stiffness and higher damping,
- Pulp/root structures are mechanically compliant and highly dissipative.

The resulting system is a chain of three coupled degrees of freedom.

**Figure 1:**
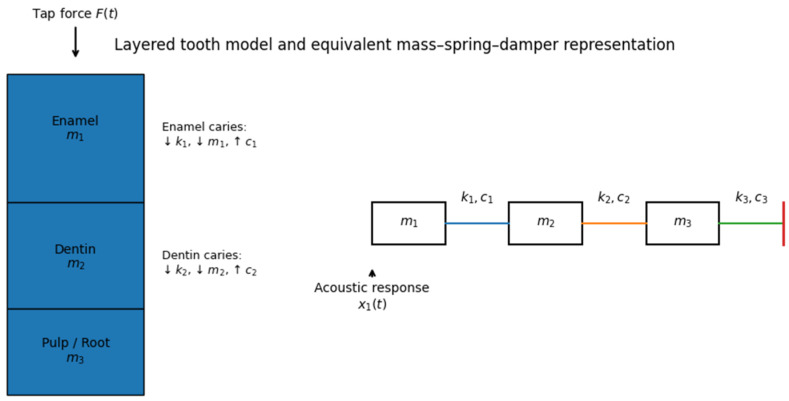
Layered tooth model and equivalent mass–spring–damper representation used in the in silico simulations. **Left**: simplified anatomical representation of enamel, dentin, and pulp/root layers, with tooth percussion modeled as an impulse force *F*(*t*) applied at the occlusal surface. **Right**: corresponding three-degree-of-freedom mass–spring–damper system, where *m*_1_, *m*_2_, *m*_3_ denote effective layer masses; *k*_1_, *k*_2_, *k*_3_ inter-layer and support stiffnesses; and *c*_1_, *c*_2_, *c*_3_ damping coefficients. Enamel and dentin caries are simulated by localized reductions in stiffness and mass and increases in damping in the corresponding elements. The normalized displacement response of the enamel mass *x*_1_(*t*) is used as the simulated acoustic signal.

A conceptual illustration of the layered tooth representation and its equivalent mass–spring–damper model is shown in Figure 1.

It is important to emphasize that this representation constitutes a reduced-order mechanical abstraction rather than a high-fidelity anatomical model. The one-dimensional, three-degree-of-freedom lumped mass–spring–damper system is intended to isolate fundamental relationships between structural parameter changes and impulse-response behavior. It does not capture full three-dimensional geometry, anisotropy, periodontal ligament dynamics, bone coupling, or complex boundary conditions present in vivo. Consequently, the simulated signals should be interpreted as idealized responses suitable for feasibility exploration rather than as realistic clinical acoustic waveforms.

#### 2.2.2. Governing Equations of Motion

The dynamics of the system were governed by the second-order matrix differential equation:

(1) $$M\ddot{x}\left(t\right)+C\dot{x}\left(t\right)+Kx\left(t\right)=F(t)$$

where

- $x\left(t\right)={\left[x_{1}\left(t\right),x_{2}\left(t\right),x_{3}(t)\right]}^{⊺}$ denotes layer displacements,
- **M** is the mass matrix,
- **C** is the damping matrix,
- **K** is the stiffness matrix,
- **F**(*t*) is the external percussion force.

#### 2.2.3. Mass, Stiffness, and Damping Matrices

The mass matrix was defined as:

(2) $$M=\left[\begin{array}{ccc} m_{1} & 0 & 0\\ 0 & m_{2} & 0\\ 0 & 0 & m_{3} \end{array}\right]$$

The stiffness matrix captured inter-layer elastic coupling:

(3) $$K=\left[\begin{array}{ccc} k_{1} & -k_{1} & 0\\ -k_{1} & {k_{1}+k}_{2} & -k_{2}\\ 0 & -k_{2} & {k_{2}+k}_{3} \end{array}\right]$$

The damping matrix was defined analogously:

(4) $$C=\left[\begin{array}{ccc} c_{1} & -c_{1} & 0\\ -c_{1} & {c_{1}+c}_{2} & -c_{2}\\ 0 & -c_{2} & {c_{2}+c}_{3} \end{array}\right]$$

This formulation ensures physically consistent force transmission and energy dissipation across layers.

#### 2.2.4. Baseline Mechanical Parameters

Baseline “healthy” parameter values were selected to produce stable and physically consistent impulse responses within the reduced-order model. These values were not calibrated against patient-specific empirical measurements, but were chosen to reflect qualitative mechanical differences between enamel, dentin, and pulp/root tissues (e.g., relatively higher stiffness and lower damping for enamel compared with dentin) (see Table 1).

The absolute numerical values should therefore be interpreted as illustrative rather than as validated biomechanical constants. To mitigate parameter subjectivity, all simulations incorporate random perturbations of baseline parameters, and robustness experiments systematically increase inter-tooth variability. This approach allows assessment of classification stability across a range of plausible parameter configurations within the simplified modeling framework.

To reflect inter-tooth variability, all parameters were randomly perturbed by ±5% for each simulated sample.

### 2.3. Tooth Percussion Simulation

#### 2.3.1. Impulse Force Modeling

Tooth percussion was modeled as a short-duration force impulse applied to the enamel layer:

(5) $$F\left(t\right)=\left\{\begin{array}{c} {\left[F_{0},0,0\right]}^{⊺},\;\;\;0\leq t\leq \tau \\ 0,\;\;\;\;\;\;\;\;\;\;\;\;\;\;\;\;\;\;\;\;\;\;\;\;\;t>\tau \end{array}\right.$$

where:

- *F*_0_ = 1.0 (arbitrary units),
- *τ* = 0.1 ms.

This formulation approximates a controlled tap at the occlusal surface.

The impulse amplitude and duration were fixed for baseline simulations to isolate structural response effects within the simplified model. No variability in tapping force, contact duration, or impact location was introduced at this stage. This design choice enables controlled feasibility assessment but does not reflect the variability expected in real clinical percussion, where operator technique and contact mechanics introduce substantial excitation variability.

Because signals are subsequently normalized by maximum amplitude (Section 2.5), global scaling effects due to impulse magnitude are largely suppressed in the feature representation; however, variability in excitation dynamics (e.g., contact duration or nonlinear impact behavior) is not explicitly modeled.

#### 2.3.2. Numerical Integration

Equation (1) was transformed into a first-order state-space system and solved numerically using an explicit Runge–Kutta method (RK45). Initial conditions were zero displacement and velocity for all layers.

Simulation parameters:

- Sampling rate: 44.1 kHz,
- Total duration: 50 ms,
- Number of samples: 2205.

### 2.4. Caries Modeling

#### 2.4.1. Structural Degradation Model

Caries were modeled as localized structural degradation affecting specific layers. A lesion severity factor α ∈ [0,1] was introduced, modifying mechanical parameters as:

(6) $$k_{i}^{caries}=k_{i}\left(1-\beta_{k,i}\alpha \right),\;\;\;\;\;\;c_{i}^{caries}=c_{i}\left(1+\beta_{c,i}\alpha \right),\;\;\;\;\;\;m_{i}^{caries}=m_{i}(1-\beta_{m,i}\alpha )$$

Parameter scaling coefficients were chosen to reflect the qualitative mechanical effects of demineralization, namely reduced stiffness and effective mass and increased damping, with stronger effects for deeper lesions. In the present study, enamel caries were modeled using $\beta_{k,1}=0.6,\;\beta_{c,1}=0.8$ and $\beta_{m,1}=0.3$, while dentin caries were modeled using $\beta_{k,2}=0.8,\;\beta_{c,2}=1.0$ and $\beta_{m,2}=0.3$. Parameters of unaffected layers were left unchanged. These coefficients were selected to explore structured, depth-dependent mechanical degradation within the simplified framework and were not calibrated to empirical material-property measurements. Accordingly, the modeled caries effects should be interpreted as parametric perturbations designed to test whether systematic structural changes could, in principle, produce separable impulse-response patterns, rather than as quantitatively validated representations of in vivo demineralization.

#### 2.4.2. Caries Classes

Three screening-relevant classes were defined:

- Healthy: no parameter modification,
- Enamel caries: modifications applied to *k*_1_, *c*_1_, *m*_1_,
- Dentin caries: stronger modifications applied to *k*_2_, *c*_2_, *m*_2_.

The use of partially overlapping but shifted severity ranges introduces structured differences between enamel and dentin classes within the simulator. This modeling choice reflects the assumption that deeper lesions produce larger mechanical perturbations; however, it may also contribute to class separability. Accordingly, results should be interpreted as demonstrating discriminative trends within this structured parameterization rather than as evidence that equivalent separation would necessarily arise under fully overlapping severity distributions.

Lesion severity was sampled from:

- Enamel caries: α ∈ [0.2, 0.5],
- Dentin caries: α ∈ [0.4, 0.8].

It is acknowledged that the choice of severity ranges and layer-specific parameter modifications may influence class separability within the simulator. The objective of this modeling strategy is not to guarantee separation, but to introduce structured and progressively stronger mechanical perturbations consistent with shallower (enamel) versus deeper (dentin) degradation. The presence of overlapping feature distributions and performance degradation under increased variability suggests that separability is not trivially hard-coded, but reflects multivariate trends within the imposed structural perturbations. Because dentin caries involve deeper-layer perturbations with larger effective parameter modifications, this class may be structurally more separable within the simulator than enamel caries, independent of clinical detectability.

### 2.5. Signal Generation and Noise Modeling

The simulated percussion-response signal was defined as the normalized displacement of the enamel layer:

(7) $$s\left(t\right)=\frac{x_{1}(t)}{\underset{t}{\max}\left|x_{1}(t)\right|}$$

Per-sample normalization by the maximum absolute displacement removes global amplitude scaling effects attributable to impulse magnitude and overall stiffness variation within the simplified model. This design choice emphasizes relative temporal decay and spectral structure rather than absolute amplitude differences. We acknowledge that normalization strategies can influence class separability; accordingly, the reported performance should be interpreted as reflecting structural pattern discrimination under controlled scaling assumptions, rather than amplitude-driven classification.

In this study, *s*(*t*) is interpreted as a proxy for a measurable acoustic or vibrational waveform rather than as a fully modeled sound pressure signal. Previous studies in dentistry have demonstrated that percussion sound frequency and tooth vibration characteristics can reflect underlying mechanical properties and clinical conditions, supporting the plausibility of vibration-based signal analysis in oral tissues [18,19]. Conceptually, *s*(*t*) approximates the surface vibration that could be sensed by a contact accelerometer or a miniature contact microphone placed on the enamel, with the understanding that real sensors would apply their own transfer functions, frequency-dependent gains, and noise characteristics.

Importantly, the present model does not include an explicit acoustic radiation model, sensor transfer-function modeling, coupling variability, or environmental acoustic effects. The simulated waveform therefore represents the underlying structural vibration component only, not the complete transduction chain from mechanical excitation to clinically recorded acoustic measurement. As such, the reported results should be interpreted as feasibility within a structural vibration framework rather than as a direct simulation of real-world acoustic acquisition.

Additive measurement noise was applied:

(8) $$\tilde{s}\left(t\right)=s\left(t\right)+\epsilon \left(t\right),\;\;\;\;\epsilon (t)\sim N(0,\sigma^{2})$$

with baseline σ = 0.01. This additive Gaussian noise term represents a minimal stochastic perturbation and does not model structured environmental noise, sensor nonlinearity, or operator-dependent variability. It serves as a controlled stress test within the simplified simulation framework rather than as a realistic clinical noise model.

All normalization and noise perturbation operations are performed independently for each simulated sample prior to dataset assembly and train–test splitting, ensuring that no dataset-level statistics are shared across subsets.

### 2.6. Dataset Generation

A total of 1500 audio samples were generated (500 per class). Each sample was saved as a WAV file, and metadata including class label, lesion type, and lesion severity were stored in a structured CSV file. Each simulated sample corresponded to an independent draw of the full parameter set, including baseline mechanical properties, lesion severity, and measurement noise. No parameter sets were reused across samples. Consequently, all samples can be interpreted as representing distinct synthetic teeth, and standard stratified train/test splitting does not introduce information leakage.

### 2.7. Feature Extraction

Mel-frequency cepstral coefficients (MFCCs) were extracted from each signal using:

- 13 coefficients,
- 512-point FFT,
- 40 Mel filters.

MFCCs were averaged across time frames to yield fixed-length feature vectors.

### 2.8. Classification and Robustness Experiments

A random forest classifier with 200 trees was implemented for screening-level classification. For baseline experiments, the full dataset was partitioned using a stratified 80% training/20% testing split to preserve class proportions. No cross-validation was used for hyperparameter optimization; instead, model hyperparameters were fixed a priori (number of trees = 200; all other parameters at default settings) to focus on feasibility assessment rather than performance maximization. To assess stability with respect to data partitioning and random initialization, the entire training–testing procedure was repeated five times using different random seeds. Random seeds were explicitly set to control dataset shuffling, train–test partitioning, parameter perturbation, noise generation, and model initialization. This ensured reproducibility across repeated runs while maintaining independence between training and testing subsets. Performance was evaluated using accuracy and macro-averaged F1-score.

Robustness experiments systematically varied:

- Noise level (σ = 0.01 to 0.03),
- Parameter variability (±5% to ±10%).

For robustness experiments, each configuration was evaluated over five independent runs with different random seeds, and mean ± standard deviation performance was reported. Baseline class-wise metrics (Table 2) are shown for a representative stratified split; averaged baseline performance is reported in Table 3 for consistency.

Because the objective is feasibility assessment within a synthetic framework, evaluation focused on overall accuracy and macro-averaged F1-score to capture balanced multi-class performance, rather than deployment-oriented metrics such as calibration curves or decision-threshold analysis.

These procedures were implemented to reduce potential sources of methodological bias within the synthetic pipeline, including partition-dependent performance inflation, parameter reuse, and uncontrolled randomness.

As the study was entirely simulation-based and did not involve human participants or subjective outcome assessment, blinding in the conventional clinical sense was not applicable. Randomization was incorporated at multiple levels of the synthetic pipeline, including parameter perturbation, lesion severity sampling, dataset shuffling, and train–test partitioning through controlled random seeds.

### 2.9. Visualization and Sanity Checks

**Figure 2:**
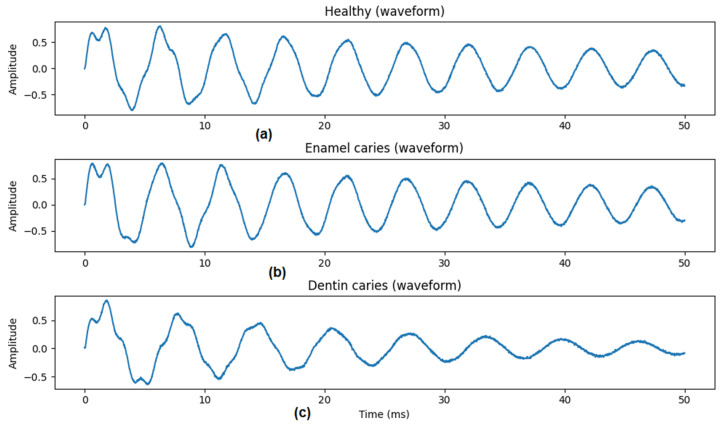
Simulated tooth percussion signals (time domain). Representative time-domain acoustic responses generated by the in silico tooth percussion model for (**a**) healthy teeth, (**b**) enamel caries, and (**c**) dentin caries. Signals correspond to the normalized displacement response of the enamel layer following a short impulse excitation. Compared with healthy teeth, carious conditions exhibit altered decay characteristics and oscillation patterns, reflecting changes in underlying mechanical properties. These differences motivate the use of acoustic features for screening-level discrimination.

**Figure 3:**
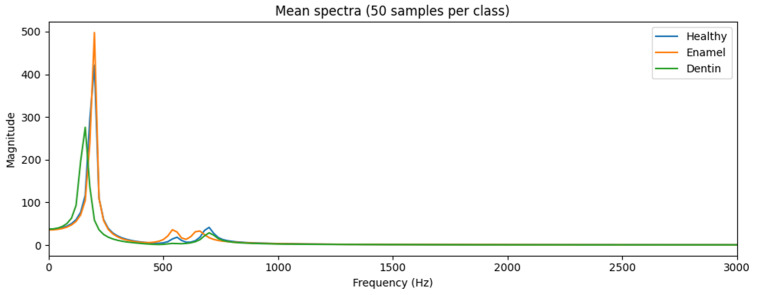
Frequency-domain characteristics of simulated signals. Mean frequency spectra computed from simulated percussion signals for healthy teeth, enamel caries, and dentin caries (averaged over multiple samples per class). Structural degradation associated with caries leads to systematic shifts in spectral energy distribution and attenuation, particularly at higher frequencies. Although individual spectra overlap, class-dependent trends are observable, supporting the feasibility of multivariate classification approaches.

**Figure 4:**
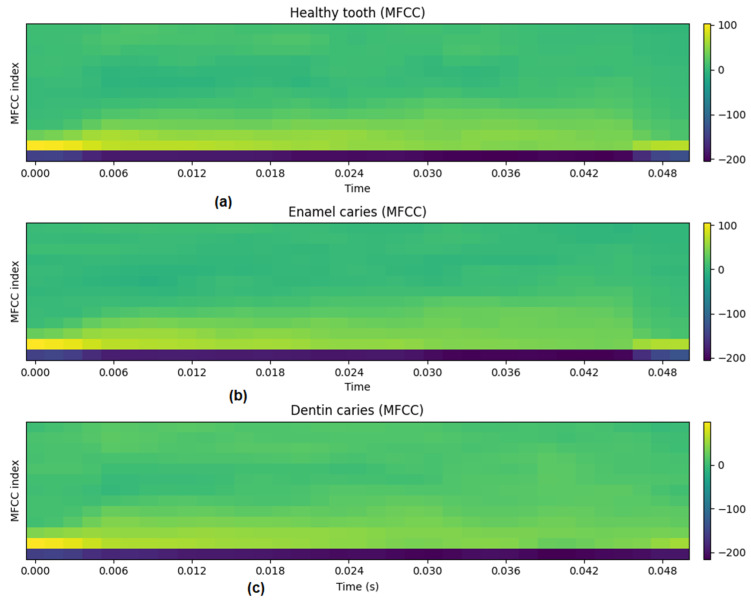
Mel-frequency cepstral coefficient (MFCC) representations. Mel-frequency cepstral coefficient (MFCC) representations of representative simulated percussion signals for (**a**) healthy teeth, (**b**) enamel caries, and (**c**) dentin caries. MFCCs summarize spectral content over time in a compact form commonly used in acoustic analysis. While individual coefficients show partial overlap across classes, their combined patterns capture subtle but systematic differences induced by caries-related structural changes.

The following plots were generated to verify physical plausibility:

- Figure 2: Time-domain waveforms for each class,
- Figure 3: Mean frequency spectra (50 samples per class),
- Figure 4: MFCC representations for representative samples.

These visualizations confirmed class-dependent differences in decay and spectral content.

**Figure 5:**
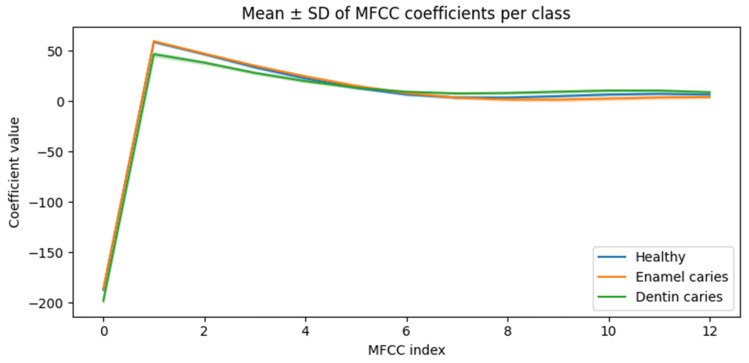
Distribution of MFCC features across classes. Mean MFCC values aggregated across all simulated samples for each class, illustrating overlap and separation in the feature space. Shaded regions (where applicable) represent variability across samples. Although no single coefficient provides complete separation, the multivariate feature structure enables effective screening-level classification, as demonstrated by machine learning results.

The visualizations in Figure 2, Figure 3, Figure 4 and Figure 5 are intended as qualitative plausibility checks to illustrate signal behavior and feature distributions rather than as formal separability analyses. Quantitative discrimination performance is evaluated through the classification metrics reported in Section 3.

### 2.10. Ethical Considerations

All data were synthetically generated. No human participants, extracted teeth, biological specimens, or in vivo recordings were involved. Therefore, ethical approval and informed consent were not required.

## 3. Results

### 3.1. Simulated Tooth Percussion Signals

The in silico tooth percussion model generated stable and physically plausible transient responses across all simulated conditions. Representative time-domain signals for healthy teeth, enamel caries, and dentin caries are shown in Figure 2. All signals exhibited a damped oscillatory response following impulse excitation, with decay characteristics varying according to underlying structural parameters.

Compared with healthy teeth, simulated carious conditions produced responses with altered amplitude decay and oscillation patterns. Enamel caries generally resulted in moderate changes to signal decay, whereas dentin caries produced more pronounced attenuation and temporal smoothing. These differences were consistently observed across randomly generated samples, indicating that the model captures systematic effects of structural degradation.

### 3.2. Frequency-Domain Characteristics

Frequency-domain analysis revealed class-dependent differences in spectral content. Mean frequency spectra averaged across multiple samples per class are presented in Figure 3. Healthy teeth exhibited higher-frequency components with slower spectral roll-off, whereas carious conditions showed increased attenuation, particularly at higher frequencies.

Dentin caries produced the most pronounced spectral changes, consistent with deeper structural involvement. Although substantial overlap between classes was observed at individual frequency bins, the overall spectral profiles differed in a systematic manner, suggesting that discriminative information is distributed across the spectrum rather than concentrated at a single frequency.

### 3.3. MFCC Feature Representation

Mel-frequency cepstral coefficients (MFCCs) were extracted from all simulated signals to provide a compact acoustic representation. Representative MFCC maps for each class are shown in Figure 4. Visual inspection indicated partial overlap between classes, with no single coefficient providing clear separation.

Aggregate MFCC statistics across the full dataset are summarized in Figure 5, which shows mean coefficient values and variability for each class. While individual MFCC dimensions exhibited overlapping distributions, consistent shifts in multiple coefficients were observed between healthy and carious conditions, particularly for dentin caries. These results indicate that discriminative information is embedded in the multivariate MFCC feature space.

Although individual MFCC dimensions exhibit overlapping distributions, multivariate separability is quantitatively reflected in the classification performance reported in Section 3.4, indicating that discriminative information emerges from joint feature structure rather than single-coefficient differences.

### 3.4. Baseline Classification Performance

Using MFCC mean features and a random forest classifier, screening-level classification performance was evaluated on a held-out test set. Under baseline conditions, characterized by low measurement noise and moderate inter-tooth variability, the classifier achieved an overall accuracy of 0.97 and a macro-averaged F1-score of 0.97 across three screening classes (healthy teeth, enamel caries, and dentin caries). Class-wise precision, recall, and F1-score values are summarized in Table 2.

Baseline class-wise metrics shown in Table 2 correspond to a representative stratified split; mean ± SD across repeated runs are reported in Table 3 for aggregate performance stability.

To provide additional quantitative insight into feature contribution, random forest feature importance analysis indicated that discriminative power was distributed across multiple MFCCs rather than concentrated in a single dominant dimension, supporting the interpretation of multivariate separability.

The corresponding confusion matrix for this representative baseline split is provided in Supplementary Table S1.

Class-specific performance was balanced, with high precision and recall observed for all categories. Misclassifications occurred primarily between healthy and enamel caries samples, whereas dentin caries were consistently identified with high accuracy. These results demonstrate that the simulated acoustic signals contain sufficient information to support screening-level discrimination under idealized conditions.

From an interpretive standpoint, these performance levels reflect screening-level separability within the defined simulation framework rather than diagnostic certainty. The stronger separation observed for dentin caries is consistent with the deeper and larger parameter perturbations imposed in the model, which structurally differentiate this class more strongly than enamel lesions. Consequently, the dentin performance should be viewed as a property of the imposed parameterization rather than as direct evidence of equivalent clinical detectability.

Random forest feature importance analysis (Supplementary Figure S2) indicated that discriminative contribution was distributed across multiple MFCCs rather than dominated by a single dimension. While higher-index coefficients (e.g., MFCC 10–12) exhibited comparatively greater importance, meaningful contribution was observed across several coefficients, supporting the interpretation that separability arises from multivariate feature structure rather than isolated single-feature effects.

### 3.5. Robustness to Noise and Parameter Variability

**Figure 6:**
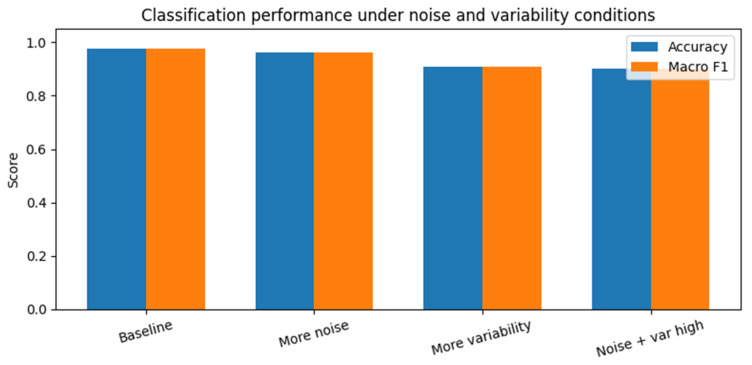
Classification performance under increasing noise and variability. Classification accuracy obtained under progressively more challenging simulation conditions, including increased measurement noise, increased inter-tooth parameter variability, and their combination. Performance degrades gradually as uncertainty increases, remaining well above chance level. This behavior indicates robustness and supports the potential use of the proposed approach as a decision-support screening tool under realistic operational conditions.

To evaluate robustness under more challenging conditions, classification experiments were repeated with increased measurement noise and increased inter-tooth parameter variability. Results are summarized in Figure 6 and Table 3. Because each robustness condition was evaluated over five independent runs, performance dispersion is quantitatively reported as mean ± standard deviation in Table 3; this numerical summary fully captures variability across repeated experiments.

As shown in Table 3, increasing the measurement noise standard deviation from 0.01 to 0.03 resulted in a modest reduction in mean classification accuracy from 0.977 to 0.967. Increasing inter-tooth parameter variability from ±5% to ±10% produced a larger decrease in mean accuracy, which declined to 0.916. When both noise and parameter variability were increased simultaneously, the mean classification accuracy decreased further to 0.903, with a corresponding macro-averaged F1-score of 0.903.

To assess sensitivity to random initialization and train–test partitioning, each robustness configuration was repeated five times using different random seeds. Across all conditions, the standard deviation of accuracy and macro-averaged F1-score remained below 0.016, indicating that the observed performance trends are stable and not driven by a favorable random split or initialization.

Despite these reductions, performance remained well above chance level across all tested conditions. The gradual degradation observed across scenarios indicates that the proposed screening approach is not overly sensitive to moderate levels of uncertainty within the bounds of the in silico model.

These experiments partially address parameter-selection subjectivity by demonstrating that classification performance degrades gradually rather than collapsing under expanded variability ranges within the model.

The robustness experiments presented here should be interpreted as internal stress tests within the same simulation framework rather than as evidence of external robustness. All perturbations—noise increases and parameter variability—were applied within the defined model structure and therefore assess sensitivity to controlled deviations from baseline assumptions. These experiments do not simulate independent acquisition conditions, alternative sensing modalities, or clinically observed variability beyond the imposed parameter ranges.

### 3.6. Summary of Quantitative Results

A summary of classification performance across all experimental conditions is provided in Table 3. Together, the results demonstrate that:

1. Caries-related structural changes produce measurable differences in simulated percussion acoustics.
2. These differences are not trivially separable in single dimensions but become discriminative when considered jointly.
3. Screening performance degrades smoothly under increased noise and variability, indicating robustness.

From a healthcare operations perspective, these quantitative findings suggest that the proposed screening approach could support practical outcomes such as the reduction in unnecessary radiographic examinations, earlier identification of higher-risk patients, and smoother patient flow through diagnostic pathways. Even imperfect screening performance may yield system-level benefits by filtering low-risk cases and reserving resource-intensive diagnostics for patients most likely to benefit.

Because all experiments are conducted within the same simplified simulation framework, the reported performance should be interpreted as an internal feasibility estimate and likely represents an upper bound relative to expected real-world performance.

The reported metrics quantify internal separability within the simulation environment and do not constitute clinically calibrated performance estimates. Threshold-dependent measures such as sensitivity/specificity trade-offs or calibration performance would require empirical validation with real-world data.

Accordingly, the observed gradual degradation under increased variability reflects model-internal sensitivity rather than validated generalization to independent real-world conditions.

## 4. Discussion

The following discussion interprets the in silico findings within a healthcare management context. However, it is important to clarify that the present study does not include formal operational research methods such as workflow modeling, triage utility curve analysis, resource-allocation simulation, or cost-effectiveness evaluation. The managerial implications discussed below are therefore conceptual and intended to outline potential directions for future implementation-oriented research, rather than to represent demonstrated operational impact.

This study evaluated the feasibility of an acoustic, machine-learning-based screening approach for dental caries from a healthcare management perspective, using an in silico modeling framework. Rather than proposing a replacement for established diagnostic procedures, the findings should be interpreted in the context of decision support, workflow optimization, and resource-efficient care delivery. The results demonstrate that caries-related structural changes can be detected from simulated tooth percussion responses with high accuracy under idealized conditions and with graceful degradation under increased noise and anatomical variability. These observations have several implications for cost management, clinical workflow design, and technology adoption in dental care systems.

### 4.1. Cost and Resource Implications

From a cost-management standpoint, dental radiography represents a significant component of diagnostic expenditure, encompassing equipment acquisition, maintenance, regulatory compliance, and trained personnel requirements [3,4,5]. While radiographic imaging remains essential for definitive diagnosis, its routine use in screening and preventive contexts can contribute to inefficiencies, particularly in high-throughput practices and public health programs. The proposed acoustic screening approach, if validated clinically, could function as a low-cost front-line screening tool, enabling early risk stratification without immediate reliance on imaging.

The in silico results suggest that such an approach may be sufficiently robust to support screening decisions under variable conditions, with classification performance remaining above chance even under combined noise and anatomical variability. From a managerial perspective, these findings motivate future evaluation of whether such a screening layer could contribute to reducing unnecessary imaging, prioritizing high-risk cases, and allocating diagnostic resources more efficiently under real-world conditions. Importantly, the economic argument for such a tool does not depend on perfect accuracy; rather, its value lies in supporting early identification and referral decisions that can prevent progression to more costly interventions [2,5].

If validated in clinical settings and integrated into defined care pathways, such a screening layer could be evaluated for its potential to reduce precautionary radiographs, decrease chair time per patient, and improve utilization of imaging resources. These effects are particularly relevant in high-throughput environments, where marginal efficiency gains can accumulate into substantial cost savings at the organizational level.

It is important to emphasize that the present study does not provide a cost-effectiveness analysis or implementation budget assessment. Any economic implications discussed here should be interpreted as managerial hypotheses contingent upon successful technical validation and subsequent real-world evaluation.

### 4.2. Workflow Integration and Operational Considerations

Beyond direct costs, diagnostic technologies shape clinical workflows and influence staff utilization. Radiographic imaging requires dedicated equipment, room availability, and trained operators, often introducing scheduling constraints and bottlenecks in busy practices. In contrast, an acoustic screening approach based on tooth percussion could, in principle, be integrated into existing examination routines with minimal disruption. Because percussion is already part of standard dental assessment, augmenting this step with objective decision support may require limited additional training or infrastructure.

From an operational perspective, such a tool could support task shifting by enabling auxiliary staff, such as dental hygienists or community health workers, to perform preliminary screening and flag cases for further evaluation. This aligns with broader healthcare management strategies aimed at improving efficiency and expanding service capacity without proportionally increasing specialist workload [7,12]. The robustness analysis presented in this study further suggests that the approach may tolerate realistic variability, an important consideration for deployment across diverse clinical environments.

From an implementation perspective, the simplicity of the proposed approach is a key feasibility factor. Because tooth percussion is already embedded in routine examinations, the addition of acoustic capture and automated analysis could be achieved with limited disruption to existing workflows. This lowers barriers to adoption compared with technologies requiring new clinical procedures, specialized facilities, or extensive retraining.

### 4.3. Managerial Interpretation and Decision-Support Use Cases

From a healthcare management perspective, the value of the proposed acoustic screening approach lies not in definitive diagnosis, but in its potential role as an early decision-support layer within dental care pathways. The results presented in this study indicate that simulated tooth percussion signals contain sufficient information to support risk stratification and triage, enabling providers to distinguish low-risk from higher-risk cases at the point of initial assessment.

In practical terms, if future clinical validation confirms comparable signal separability in real-world recordings, such a screening concept could potentially contribute to decisions regarding radiographic prescription, monitoring strategies, or referral prioritization. However, the present in silico study does not evaluate decision thresholds, referral criteria, false-positive/false-negative trade-offs, or workflow performance metrics. Accordingly, the discussion of front-end triage applications should be interpreted as a conceptual managerial framework rather than as evidence of demonstrated reductions in imaging utilization, visit duration, or patient flow constraints.

Importantly, the managerial utility of this approach does not depend on perfect classification accuracy. Even moderate screening performance may yield meaningful system-level benefits by filtering out clearly low-risk cases and concentrating diagnostic resources on patients most likely to benefit. This aligns with established healthcare management principles, where decision-support tools are evaluated based on their contribution to efficiency, consistency, and resource optimization rather than individual-level diagnostic certainty.

At the organizational level, the proposed approach could support standardized screening protocols, task redistribution to auxiliary staff, and more predictable utilization of imaging infrastructure. These characteristics are especially relevant for public dental services, mobile clinics, and community-based programs, where scalability, cost control, and access to preventive care are central management objectives. However, these potential organizational effects remain hypothetical pending empirical workflow studies and cost-effectiveness analyses.

### 4.4. Comparison with Existing Approaches in the Literature

A growing body of literature has explored the application of artificial intelligence and machine-learning techniques for dental caries detection, predominantly using radiographic images or intraoral photographs as input data. Currently available caries diagnostic approaches can be broadly categorized into: (1) visual–tactile examination, (2) radiographic imaging (including AI-enhanced radiographic interpretation), and (3) adjunct non-radiographic technologies such as fluorescence-, optical-, and impedance-based systems. Many of these studies aim to automate or enhance diagnostic accuracy, often reporting high performance for lesion detection and classification [20,21,22]. Recent systematic reviews and meta-analyses confirm that contemporary AI applications for caries detection are overwhelmingly imaging-based, relying primarily on radiographic or photographic inputs rather than alternative physical sensing modalities [23,24,25,26]. While such approaches demonstrate clear technical promise, they typically rely on resource-intensive imaging modalities and are designed to support or replicate diagnostic decision-making, rather than to assist front-end screening, triage, or resource allocation.

In contrast, the present study addresses a different problem space. Rather than focusing on diagnostic automation, the proposed approach is positioned as a low-cost, non-invasive screening and decision-support tool intended to complement existing workflows. The use of acoustic tooth percussion signals distinguishes this work from imaging-based methods and aligns it more closely with managerial objectives such as cost control, workflow efficiency, and scalability in resource-constrained settings. This distinction is consistent with broader perspectives in healthcare AI, which emphasize decision support and augmentation of clinical workflows over full diagnostic replacement [13].

Prior research has also investigated non-radiographic methods for caries assessment, including optical, fluorescence-based, and electrical impedance techniques [27,28,29]. While these approaches reduce radiation exposure, they often require specialized hardware, controlled acquisition conditions, or additional clinical steps, which may limit their applicability in high-throughput or decentralized care environments. By leveraging a routine clinical action (tooth percussion) and minimal sensing requirements, the proposed acoustic approach prioritizes feasibility, integration, and scalability over lesion-specific diagnostic precision. It is important to note that direct precedent for percussion-derived acoustic analysis specifically targeting caries depth classification is limited in the existing literature. Tooth percussion has traditionally been used qualitatively in endodontic assessment and mobility testing rather than for structural lesion staging. The present study therefore does not build upon a mature acoustic caries-staging literature, but instead explores a previously underexamined hypothesis: that depth-dependent mineral loss may measurably alter transient vibrational characteristics. The plausibility of this hypothesis is grounded in established principles of vibration mechanics and material degradation rather than in an existing body of caries-specific acoustic validation studies. Accordingly, the contribution of this work is exploratory and hypothesis-generating rather than confirmatory within an established acoustic diagnostic paradigm.

Finally, in silico modeling has been increasingly recognized as a valuable tool for early-stage evaluation of healthcare technologies, particularly when clinical data collection is costly, time-consuming, or ethically complex [15,16,30]. Compared with data-driven studies relying exclusively on curated clinical datasets, the present in silico feasibility analysis enables systematic exploration of variability and robustness under controlled conditions. In this sense, the contribution of this work lies not in outperforming existing diagnostic systems, but in defining and evaluating a complementary screening paradigm aligned with healthcare system needs and managerial decision-making requirements.

### 4.5. Clinical Context and Integration into Diagnostic Pathways

From a clinical perspective, the proposed acoustic screening approach is intended to function as an adjunct to, rather than a replacement for, established diagnostic procedures. Its role is best understood in relation to existing caries detection pathways, which typically combine visual–tactile examination with selective use of bitewing radiographs based on patient risk and clinical findings.

In routine dental practice, bitewing radiographs are not obtained at every visit for every patient, but are prescribed according to guideline-based recall intervals and perceived caries risk. Within this framework, the acoustic screening tool would be applied prior to radiographic imaging during the chairside examination. A low-risk screening result could support the clinician’s decision to defer radiographs when clinically appropriate, whereas a higher-risk result could prompt earlier or more targeted radiographic assessment.

Because the proposed system is a screening tool, its clinical performance requirements differ from those of a diagnostic test. In particular, some level of false-negative results is clinically acceptable, provided that such errors do not exceed the limitations of routine visual–tactile examination or result in delayed detection of lesions likely to progress within standard recall intervals. Missed early enamel lesions that remain detectable through follow-up examinations, preventive monitoring, or guideline-based imaging would therefore represent an acceptable trade-off in a screening context.

It is important to clarify that the three classes used in the present in silico framework (healthy, enamel caries, dentin caries) represent simplified structural depth categories rather than clinically validated management states. In real dental practice, lesion activity (active vs. arrested), cavitation status, anatomical location (occlusal vs. proximal), patient-specific risk factors, and restorative history all influence treatment decisions. The simulator does not model these dimensions, and therefore the class labels should not be interpreted as direct proxies for intervention thresholds. Instead, they serve as controlled structural perturbations to test whether depth-dependent mechanical degradation produces discriminative signal patterns. Translation to clinically meaningful screening categories would require future studies explicitly linking acoustic features to validated diagnostic criteria and management pathways.

Responsibility for interpreting and acting on the screening output would remain entirely with the dental professional performing the examination. The screening result is intended to complement clinical judgment, patient history, and established risk assessment rather than to dictate clinical decisions. In practice, the output could be reviewed by a dentist or dental hygienist at the chairside to inform decisions regarding radiographic prescription, preventive counseling, or monitoring strategies. Importantly, no irreversible clinical action would be taken on the basis of the screening output alone.

By explicitly situating the proposed approach within existing diagnostic pathways, its potential value can be understood as supporting earlier and more consistent risk stratification while preserving the central role of clinician judgment and confirmatory imaging. Determining appropriate sensitivity thresholds and acceptable false-negative rates will require clinical validation studies aligned with guideline-based care and patient safety considerations.

### 4.6. Limitations of In Silico Acoustic Modeling

The findings of this study must be interpreted in light of the substantial abstraction inherent in the reduced-order mechanical model. While the present framework enables systematic feasibility assessment under controlled and reproducible conditions, simulated signals can only approximate the complexity of real-world tooth percussion responses. Consequently, the reported classification performance should be regarded as an upper bound rather than an estimate of expected clinical accuracy.

The robustness analysis should therefore be viewed as assessing structural stability within the simulator rather than demonstrating external generalization across independent acquisition environments.

First, biological variability is simplified in the current model. Although inter-tooth variability was partially addressed through random perturbation of mechanical parameters, real teeth exhibit substantially greater heterogeneity in morphology, mineralization, age-related changes, restorative history, and pathological conditions beyond caries. These factors may influence acoustic responses in ways that are not fully captured by a reduced-order lumped-parameter representation. Beyond generalized biological variability, several clinically prevalent conditions may introduce signal perturbations that exceed those associated with early caries-related demineralization. These include direct and indirect restorations (composite fillings, amalgam, crowns), fissure sealants, enamel cracks, endodontically treated teeth, variations in occlusal contact patterns, tooth-type-dependent morphology (e.g., molars versus incisors), and differences in periodontal support or mobility. Many of these factors alter mass distribution, boundary conditions, and damping characteristics in ways that may produce vibrational signatures comparable to or larger than those induced by localized caries. The present in silico framework does not model these confounders and therefore does not claim that caries-related signatures would dominate in heterogeneous clinical populations. Instead, the study isolates depth-dependent structural degradation as a controlled perturbation to determine whether a discriminative signal component exists prior to the introduction of real-world complexity.

Second, enamel and dentin were modeled as isotropic layers with effective bulk mechanical properties. In reality, enamel exhibits pronounced anisotropy due to its prismatic microstructure, and dentin displays spatially varying tubule orientation and density. Such anisotropic and heterogeneous material properties can affect wave propagation, frequency-dependent attenuation, and mode coupling, potentially altering measurable acoustic signatures.

Third, the present model does not explicitly represent the periodontal ligament, alveolar bone, or surrounding soft tissues, all of which contribute to mechanical boundary conditions during tooth percussion. The periodontal ligament, in particular, introduces nonlinear compliance and damping that can significantly influence vibration transmission and decay characteristics in vivo. Neglecting these structures may lead to discrepancies between simulated and clinically observed signals.

Fourth, the simulated acoustic signal was defined as the normalized displacement response of the enamel layer, which serves as a proxy for measurable vibrations or sound pressure. In clinical practice, acoustic acquisition would depend on sensor type (e.g., microphone, accelerometer, contact transducer), coupling conditions, and placement variability. Variations in sensor contact force, orientation, and positioning relative to the tooth surface are expected to introduce additional variability not represented in the current simulations.

Finally, ambient clinical noise and operator-dependent variability were only approximated through additive Gaussian noise. Real clinical environments may introduce nonstationary noise sources, including patient movement, instrumentation sounds, and environmental acoustics, which may further degrade signal quality and classification performance. Additionally, the percussion input in the present model is idealized as a fixed-duration, fixed-amplitude impulse applied at the occlusal surface. In clinical practice, tapping force, impulse duration, contact location, and operator technique vary substantially across examinations. Such variability would introduce additional amplitude scaling, temporal variation, and nonlinear contact dynamics not represented in the current framework. Consequently, real-world excitation variability may further reduce class separability relative to the controlled impulse conditions used in this simulation. Future experimental validation should explicitly evaluate robustness to excitation variability, including operator-dependent force amplitude and contact duration distributions.

Taken together, these limitations underscore that the present in silico results should be interpreted as a feasibility demonstration rather than evidence of clinical effectiveness. Addressing these factors will require progressive validation using ex vivo tooth specimens and in vivo recordings, as well as the incorporation of more detailed biomechanical models and realistic sensing assumptions. Nevertheless, the controlled in silico framework employed here provides a necessary first step for determining whether acoustic tooth percussion contains sufficient information to justify further experimental and clinical investigation. From a sensing perspective, the relationship between the modeled enamel displacement and a clinically recorded signal can be interpreted as a transduction chain. For a contact accelerometer or contact microphone rigidly coupled to the tooth surface, the sensor output would be approximately proportional to enamel acceleration or velocity, respectively, modulated by the sensor’s frequency response and mounting conditions. For an air-coupled microphone, the relevant quantity would be the surface velocity driving sound radiation into the surrounding air, again shaped by local radiation impedance and environmental acoustics. In all cases, these transduction processes can be viewed as applying an additional, generally frequency-dependent linear filter and amplitude attenuation to the idealized displacement waveform used in this study. As a result, the real-world signal-to-noise ratio and class separability are expected to be lower than those observed in the present simulations, reinforcing the interpretation of the reported performance as an optimistic upper bound pending ex vivo and in vivo measurements.

### 4.7. Scalability and Access to Care

Access to dental care remains uneven across populations, with disparities driven by geographic, economic, and infrastructural factors [7,8]. In low-resource settings, mobile clinics, and public health screening programs, access to radiographic equipment may be limited or intermittent. Low-cost, portable screening technologies have therefore been identified as a priority for expanding preventive services and improving early detection [9,10,11].

The acoustic screening concept evaluated here aligns with these objectives by leveraging minimal hardware requirements and computational decision support. While the present study is limited to in silico evaluation, the findings suggest that further investigation into scalable implementation is warranted. From a management standpoint, such technologies could support population-level screening initiatives, reduce barriers to entry for preventive care, and inform referral pathways in settings where comprehensive diagnostics are not immediately available.

### 4.8. Adoption Barriers and Limitations

Despite its potential, several barriers to adoption must be acknowledged. First, the present study is based on simulated data and therefore represents an upper bound on achievable performance. Clinical validation using ex vivo and in vivo recordings is essential before any organizational or policy decisions can be made. Second, integration into existing care pathways would require careful consideration of liability, regulatory approval, and clinician acceptance, particularly given the sensitivity of diagnostic decision-making in dentistry [17].

Additionally, false-positive and false-negative screening outcomes have distinct managerial implications. False positives may increase referral and imaging rates, while false negatives could delay care. Future work should therefore evaluate acceptable trade-offs in screening performance based on specific organizational goals and risk tolerance. Finally, successful adoption will depend on alignment with reimbursement models, incentive structures, and local practice norms, factors that have been shown to influence the uptake of health technologies beyond their technical performance [17].

### 4.9. Clinical Significance

The clinical significance of this study lies in demonstrating, at a feasibility level, that structural changes associated with dental caries may produce measurable alterations in transient tooth vibration responses. Although the present work is limited to a controlled in silico framework, the findings suggest that routine clinical actions such as tooth percussion could potentially be augmented with objective signal analysis to support early risk stratification. If validated through ex vivo and in vivo studies, such an approach may assist clinicians in identifying cases that warrant closer monitoring or confirmatory imaging while avoiding unnecessary diagnostic interventions in clearly low-risk situations. Importantly, the proposed system is intended as a decision-support aid rather than a diagnostic replacement, preserving clinician judgment and existing standards of care.

### 4.10. Implications for Future Research and Practice

The findings of this in silico study support further exploration of acoustic screening as a decision-support mechanism in dental care management. Future research should focus on clinical validation, cost-effectiveness analysis, and pilot implementation studies to assess real-world performance and organizational impact. Such validation efforts should align with emerging regulatory frameworks for clinical AI and prospective evaluation designs that quantify real-world decision impact and workflow effects [31,32]. From a management perspective, such studies are critical for determining whether the proposed approach can meaningfully contribute to workflow optimization, cost containment, and improved access to preventive dental services.

In conclusion, this work demonstrates the feasibility of an acoustic, machine-learning-based screening approach for dental caries within a controlled in silico framework. By framing the technology as a decision-support tool rather than a diagnostic replacement, the study contributes to ongoing discussions on scalable, low-cost innovations aimed at enhancing efficiency and equity in oral health management.

## 5. Conclusions

This study presented an in silico feasibility evaluation of an acoustic, machine-learning-based screening approach for dental caries, framed explicitly as a decision-support tool rather than a diagnostic replacement.

The main findings of the study are as follows:

1. A physics-informed tooth percussion model generated simulated acoustic signals in which depth-dependent structural degradation (healthy, enamel caries, dentin caries) produced measurable differences in transient vibrational responses.
2. Screening-level classification using MFCC features and a random forest classifier achieved high baseline performance under controlled simulation conditions.
3. Robustness experiments demonstrated gradual and predictable degradation under increased measurement noise and inter-tooth parameter variability, with performance remaining well above chance within the defined model assumptions.
4. The results indicate that caries-related structural changes can be reflected in measurable acoustic responses within the simplified mechanical framework, supporting the technical feasibility of further investigation.

From a healthcare management perspective, the present findings motivate further evaluation of whether low-cost acoustic screening technologies could contribute to early risk stratification and more structured diagnostic decision pathways. However, the current in silico framework does not model radiographic utilization rates, workflow performance, cost outcomes, or real-world acquisition variability. Accordingly, the reported results should be interpreted strictly as technical feasibility evidence informing decisions about whether subsequent clinical and operational validation is warranted.

Future work will focus on:

- ex vivo and in vivo validation,
- evaluation of cost-effectiveness,
- assessment of integration into existing dental care pathways.

At the healthcare system level, the proposed screening concept illustrates a potential direction for integrating low-cost decision-support tools into dental workflows. If future empirical validation confirms real-world performance and operational viability, such technologies could then be examined for their impact on diagnostic utilization patterns, workflow structure, and resource allocation. The present study does not demonstrate system-level efficiency, sustainability, or task redistribution outcomes; rather, it provides a technical basis for subsequent translational and operational research.

## Figures and Tables

**Table 1:** Baseline mechanical parameters for healthy tooth model.

Layer	Mass *m*	Stiffness *k* (N/m)	Damping *c* (Ns/m)
Enamel	1.0	1.0 × 10^7^	50
Dentin	1.5	5.0 × 10^6^	150
Pulp/root	2.0	2.0 × 10^7^	300

**Table 2:** Class-wise precision, recall, and F1-score for the baseline screening experiment using a random forest classifier trained on MFCC features extracted from simulated tooth percussion signals.

Class	Precision	Recall	F1-Score	Support
Healthy	0.95	0.98	0.96	93
Enamel caries	0.98	0.94	0.96	88
Dentin caries	1.00	1.00	1.00	89
Macro-average	0.97	0.97	0.97	270

**Table 3:** Classification performance under increasing measurement noise and anatomical variability. Accuracy and macro-averaged F1-score are reported as mean ± standard deviation over five independent runs with different random seeds.

Condition	Noise STD	Parameter Variability (±)	Accuracy (Mean ± SD)	Macro-F1 (Mean ± SD)
Baseline	0.01	0.05	0.977 ± 0.010	0.977 ± 0.010
More noise	0.03	0.05	0.967 ± 0.009	0.967 ± 0.009
More variability	0.01	0.10	0.916 ± 0.015	0.915 ± 0.015
Noise + variability	0.03	0.10	0.903 ± 0.013	0.903 ± 0.013

## Data Availability

The data presented in this study were generated synthetically and are not publicly archived. Data and code are available from the corresponding author upon reasonable request.

## Supplementary Materials

The following supporting information can be downloaded at https://www.mdpi.com/article/10.3390/diagnostics16040638/s1, Table S1: Confusion matrix for representative baseline stratified split; Figure S1: Graphical confusion matrix visualization; Figure S2: Random forest feature importance (MFCCs).
