# Supplementary material for: Evaluating the Managerial Feasibility of an AI-Based Tooth-Percussion Signal Screening Concept for Dental Caries: An In Silico Study
Source: Diagnostics (Basel). 2026 Feb 22;16(4):638. doi: 10.3390/diagnostics16040638 (PMC12939222; doi:10.3390/diagnostics16040638)

Supplementary Materials

Evaluating the Feasibility of a Low-Cost AI-Based Acoustic Screening Tool for Dental Caries as a Diagnostic Decision-Support System: An In Silico Study

Supplementary Table S1. Confusion Matrix for Representative Baseline Stratified Split

Confusion matrix corresponding to the representative 80%/20% stratified train–test split reported in Section 3.4 of the main manuscript. Values represent counts of samples per true and predicted class.

Actual/Predicted	Healthy	Enamel Caries	Dentin Caries
**Healthy (93)**	90	3	0
**Enamel (88)**	0	88	0
**Dentin (89)**	0	0	89

Total samples in test set: 270

Overall accuracy for this representative split: 267/270 ≈ 0.989

Note: The class-wise metrics reported in Table 2 of the main manuscript correspond to this representative split. Aggregate mean ± standard deviation values across repeated runs are reported separately in Table 3.

Supplementary Figure S1. Graphical Representation of Baseline Confusion Matrix

Confusion matrix visualization for the representative stratified split described above. Values represent raw counts of samples per class.

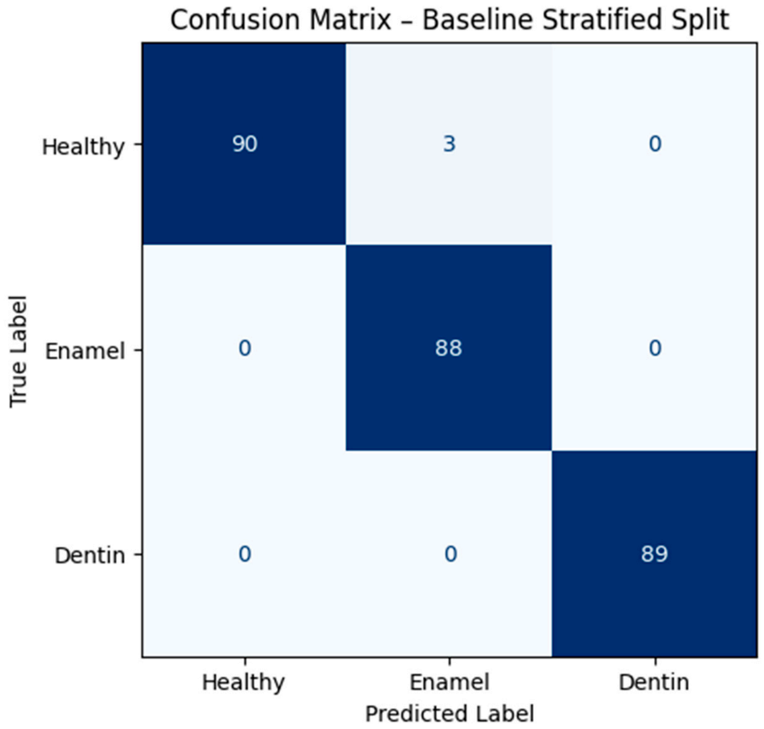

Supplementary Figure S1. Confusion matrix for representative baseline stratified split (80% training/20% testing). Rows indicate true class labels; columns indicate predicted class labels.

Reproducibility Note

The confusion matrix was generated using a stratified 80%/20% train–test split with fixed random seed (random_state = 0) and a random forest classifier with 200 trees, as described in Section 2.8 of the main manuscript. No hyperparameter optimization or cross-validation was performed for this representative baseline configuration.

Supplementary Figure S2. Random forest feature importance for MFCC coefficients (baseline stratified split).

Feature importance values derived from the trained random forest classifier (200 trees). Discriminative contribution is distributed across multiple MFCC coefficients, with higher-index coefficients showing comparatively greater influence.

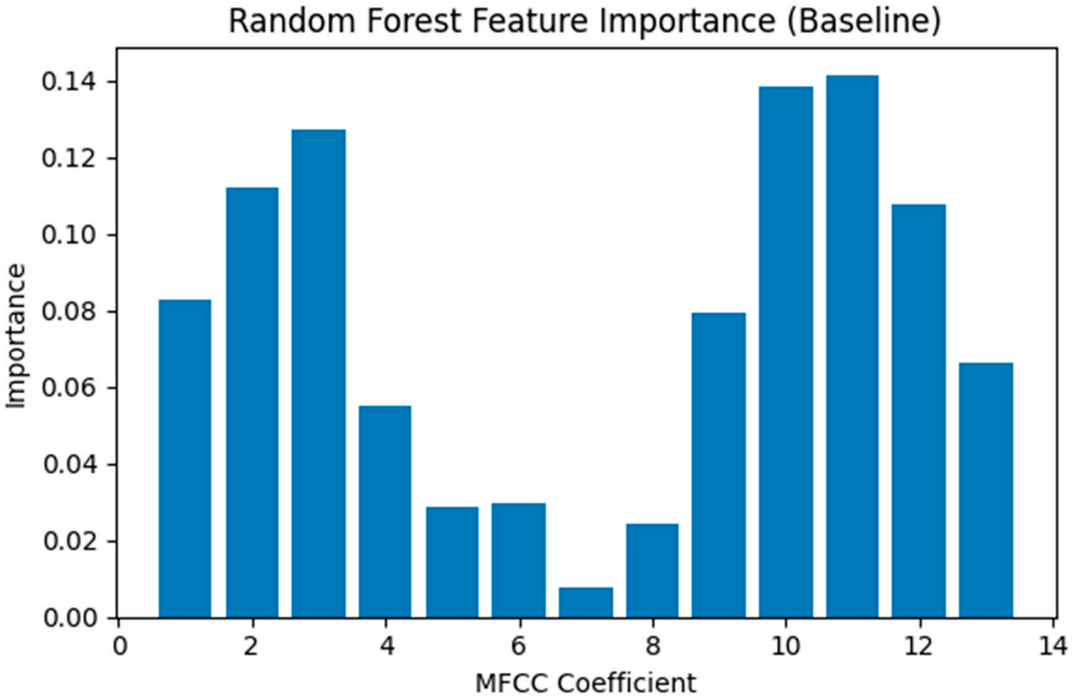

Supplement: Supplementary file 1 [file diagnostics-16-00638-s001.zip › diagnostics-4162154-supplementary.pdf]
